# Editorial: Ischemic stroke as systemic disorder involving both nervous and immune systems

**DOI:** 10.3389/fncel.2023.1208787

**Published:** 2023-05-10

**Authors:** Qingkun Liu, Yan Wang, Jui-Hung Jimmy Yen

**Affiliations:** ^1^Nash Family Department of Neuroscience, Icahn School of Medicine at Mount Sinai, New York, NY, United States; ^2^Institute of Medical Innovation and Research, Peking University Third Hospital, Beijing, China; ^3^Medical Research Center, Peking University Third Hospital, Beijing, China; ^4^Department of Microbiology and Immunology, Indiana University School of Medicine, Fort Wayne, IN, United States

**Keywords:** ischemic stroke, systemic immune responses, microglia, peripheral immune cells, biomarker for stroke diagnosis

As a systemic disorder, ischemic stroke affects millions of people worldwide with devastating consequences, including disabilities and death. Although extensive effects have been made to investigate potential clinical treatments, the time window for vascular intervention after stroke, using either thrombolytic therapy or endovascular surgery, is limited and neuroprotective approaches have failed in clinical trials. Therefore, it is critical to discover novel treatments with extended time window or to identify novel clinical biomarkers for ischemic stroke diagnosis for prompt treatments. As a multiphasic process, ischemic stroke progression is associated with long-lasting innate and adaptive immune responses, which offer appropriate time frames and targets for therapeutic interventions. To this end, this Research Topic creates a platform for researchers to (1) reveal the mechanisms of systemic immune responses and nervous system damages in ischemic stroke; and (2) discover novel biomarkers for clinical diagnosis of ischemic stroke. By including five original research articles and four comprehensive reviews, this topic covers additional critical topics in ischemic stroke, including systemic immune functions in the central nervous system and periphery, neuronal damage mechanisms, and the discovery of novel diagnostic biomarkers. These findings were categorized and summarized as follows.

The immune functions of microglia in ischemic stroke were discussed intensively in this topic. Microglia have long been taken as a main contributor to the progression of ischemic stroke. However, the dynamic functions of microglia make it challenging to revolve its exact role in different phases of ischemic stroke. In this topic, two studies investigate the mechanisms of microglial polarization in pre-clinical models. The study by Kuo et al. demonstrates a novel mechanism of IFNβ on the modulation of microglial polarization in which they identified IFNβ attenuates inflammatory and augments anti-inflammatory phenotypes of microglia in ischemic stroke mice subjected to delayed tPA treatment. They found that IFNβ-mediated modulation of microglial phenotypes plays an essential role in attenuating tPA-exacerbated ischemic brain injury and alleviating tPA-augmented BBB disruption in ischemic stroke. The study by Kong et al. showed the inhibition of stimulator of interferon genes (STING) diminishes ischemic brain infarction, edema, and neuronal injury after ischemic stroke. Mechanistically, they identified STING promotes microglial polarization toward inflammatory phenotype. In addition, a specific review of the functions and mechanisms of microglial phagocytosis is included. Jia et al. demonstrated the microglial phagocytosis as a double-sword to stroke recovery by several potential receptors sensing “eat-me” signals. On the one hand, microglia engulf live neurons and endothelial cells, resulting in excessive neuronal death and BBB leakage. On the other hand, microglia restrict inflammatory damage via engulfing cell debris, cleaning infiltrating neutrophils, and creating an optimal microenvironment for neurogenesis.

In addition to microglia, peripheral immune cells have been shown to play a systemic role in ischemic stroke. Unlike microglia, leukocytes could either infiltrate into the brain or maintain in the peripheral organs to exert both detrimental and beneficial effects on the outcomes of ischemic stroke (Benakis et al., 2016; Liu et al., 2019). In this topic, Weber et al. evaluated the transcriptomic gene changes, vascular quantification, and behavior tests among Reg2^−/−^, NSG, and tacrolimus immunosuppressed mouse as well as wild type (WT) C57BL/6J mouse stroke model. Firstly, the study indicated that the immune-deficiency mice had less macrophage infiltration and microglia activation. Secondly, although the genetic immunosuppression model is reliable for graft survival, pharmacological immunosuppression with tacrolimus could provide more accurate results with the least variation in gene expression compared to WT control group. This conclusion provides valuable baseline data for researchers to consider which mouse model would best suit for studies. In addition, a review by Zhang et al. summarized the effects and mechanisms of infiltrating peripheral immune cells, immune cell-released cytokines, and cell-cell interactions in the neurological recovery after ischemic stroke. Specifically, monocytes and macrophages actively participate in neurogenesis and oligodendrogenesis via polarization and phagocytotic function. Neutrophils can impair revascularization by releasing extracellular traps. T regulatory cells facilitate functional recovery, and B cells may regulate neurological function by antibodies. These cells and brain resident cells can also interact and communicate with each other to exert complicated effects on functional recovery.

Another topic that attracts attention is how systemic immunity involving both CNS and periphery affects ischemic stroke outcomes. The study by Weng et al. evaluated the therapeutic potential of 4-ethylguaiacol (4-EG) known as methoxyphenols in ischemic stroke. They found that 4-EG ameliorates ischemic brain injury and lessens BBB disruption in the ischemic brain. They further identified that 4-EG suppresses microglial activation, peripheral inflammatory immune cell infiltration, and brain endothelial cell adhesion molecule upregulation in the ischemic brain. These protective effects are attributed to the induction of anti-oxidant and anti-inflammatory Nrf2/HO-1 pathway. As a group of cytokines, interleukins can be expressed and secreted by both leukocytes and microglia. It plays a significant role in modulating the functions of immune cells systemically. The review by Zhu et al. divided interleukins into two categories: pro-inflammation and anti-inflammation. Another difference between these two categories is the time points they function. Specifically, pro-inflammatory IL-1 functions during the acute phase, whereas anti-inflammatory IL-10 starts to express 4 days after the brain injury. Currently, the therapeutic potential of interleukins has been well-applied in both basic and translational cancer research (Briukhovetska et al., 2021). As it has been shown, interleukins play significant roles in both acute and sub-acute phases after ischemic stroke; interleukin therapy would be an interesting and exciting future clinical research direction. Cell apoptosis, edema, endoplasmic reticulum stress (ERS), and inflammation are the main events occurring after ischemic stroke. In this topic, the review by Wang et al. summarizes many cytokines and compounds targeting ERS that show effects on alleviating ischemic brain injury.

Identifying clinical biomarkers for stroke diagnosis would benefit thousands of patients by providing prompt treatments. In this topic, Maciejczyk et al. demonstrates the potential utility of salivary xanthine oxidase (XO) in the differential diagnosis of stroke with the stress-free and non-invasive collection. In particular, XO catalyzes the generation of reactive oxygen species, which induce oxidative stress and inflammatory mediators, the leading factors for ischemic brain injury. They demonstrated that XO-specific activity in salivary distinguishes ischemic stroke from hemorrhagic stroke and healthy control with high sensitivity and specificity and therefore serves as a potential biomarker for diagnosis.

In summary, this Research Topic includes studies covering the effects of microglial polarization on ischemic brain injury outcome, the role of peripheral immune cells in the acute injury and recovery phase, roles of cytokines in modulating neuroinflammation and novel brain-protective immunomodulatory treatments. With this regard, this Research Topic contributes important findings that advance our understanding in the field of peripheral and CNS immune system interplay.

## Author contributions

QL initiated the Research Topic and drafted the editorial manuscript. YW and J-HY contribute to the writing of the editorial. All authors contribute equally to the editing of the papers in this topic. All authors contributed to the article and approved the submitted version.
